# Specialized adaptations allow vent-endemic crabs (*Xenograpsus testudinatus*) to thrive under extreme environmental hypercapnia

**DOI:** 10.1038/s41598-020-68656-1

**Published:** 2020-07-16

**Authors:** Garett J. P. Allen, Pou-Long Kuan, Yung-Che Tseng, Pung-Pung Hwang, Alex R. Quijada-Rodriguez, Dirk Weihrauch

**Affiliations:** 1 Biological Sciences, University of Manitoba, 190 Dysart Rd., Winnipeg, MB R3T 2M8 Canada; 2 Institute of Cellular and Organismal Biology’s Marine Research Station, Academia Sinica, No. 23-10 Dawen Rd., Jiaoxi, 262 Yilan County Taiwan; 3 Institute of Cellular and Organismal Biology, Academia Sinica, No. 128, Section 2, Academia Rd., Nangang District, Taipei City, 11529 Taiwan

**Keywords:** Kidney, Respiration

## Abstract

Shallow hydrothermal vent environments are typically very warm and acidic due to the mixing of ambient seawater with volcanic gasses (> 92% CO_2_) released through the seafloor making them potential ‘natural laboratories’ to study long-term adaptations to extreme hypercapnic conditions. *Xenograpsus testudinatus*, the shallow hydrothermal vent crab, is the sole metazoan inhabitant endemic to vents surrounding Kueishantao Island, Taiwan, where it inhabits waters that are generally pH 6.50 with maximum acidities reported as pH 5.50. This study assessed the acid–base regulatory capacity and the compensatory response of *X. testudinatus* to investigate its remarkable physiological adaptations. Hemolymph parameters (pH, [HCO_3_^−^], ${\text{P}}_{{{\text{CO}}_{2} }}$, [NH_4_^+^], and major ion compositions) and the whole animal’s rates of oxygen consumption and ammonia excretion were measured throughout a 14-day acclimation to pH 6.5 and 5.5. Data revealed that vent crabs are exceptionally strong acid–base regulators capable of maintaining homeostatic pH against extreme hypercapnia (pH 5.50, 24.6 kPa ${\text{P}}_{{{\text{CO}}_{2} }}$) via HCO_3_^−^/Cl^−^ exchange, retention and utilization of extracellular ammonia. Intact crabs as well as their isolated perfused gills maintained ${\text{P}}_{{{\text{CO}}_{2} }}$tensions below environmental levels suggesting the gills can excrete CO_2_ against a hemolymph-directed ${\text{P}}_{{{\text{CO}}_{2} }}$ gradient. These specialized physiological mechanisms may be amongst the adaptations required by vent-endemic animals surviving in extreme conditions.

## Introduction

Hydrothermal vents produce some of the most challenging environmental conditions found throughout the world’s oceans—often being considered the “edge of life”. As such, vent endemic animals are believed to be well-adapted to stressors such as extreme temperatures, acidities, hypoxia or anoxia, pressures, and presence of toxic compounds (i.e. sulfur, metals) driven by the cooling of magma below the oceanic floor^1^. Shallow vent systems have recently been described as natural laboratories to study CO_2_ perturbations related to predicted future ocean acidification scenarios^2–4^; particularly concerning adaptations of vent-endemic animals whom were essentially born into conditions similar to projected future oceanic acidities and temperatures. Climate change projections suggest that by the year 2300 ocean surface waters could warm by approximately 5 °C^5^ while increasing partial pressure of CO_2_ (${\text{P}}_{{{\text{CO}}_{2} }}$) simultaneously reduces oceanic pH as much as 0.7–0.8 units^6–8^. Experimental exposure of marine organisms to similar or milder conditions have demonstrated that marine organisms experience a breadth of physiological responses spanning structural, physiological, and chemosensory challenges^9^. Heightened environmental ${\text{P}}_{{{\text{CO}}_{2} }}$ tensions cause an even greater increase in an organism’s extracellular and intracellular ${\text{P}}_{{{\text{CO}}_{2} }}$_,_ as animals are generally believed to rely upon diffusive loss of metabolic CO_2_ from the cellular level to the extracellular space and eventual excretion to the environment^10,11^. Elevated arterial ${\text{P}}_{{{\text{CO}}_{2} }}$ promotes acidification of extracellular fluids, forcing organisms to either compensate or succumb to the disturbance should enzyme/protein, and thus cellular, function fail^12,13^.

Acid–base compensation is typically achieved via accumulation of HCO_3_^−^ within the extracellular fluid of an organism; however, the source of HCO_3_^−^ may be environmental (i.e. branchial HCO_3_^−^/Cl^−^ exchange^14,15^) or mobilized from stored sources and calcified structures (i.e. bones or the haemocoel and shell of calcified invertebrates)^10,16,17^. Non-bicarbonate buffers such as ammonia, proteins/amino acids, and phosphates generally play an important, but lesser role depending on species^18,19^. These compensatory mechanisms are becoming increasingly important to aquatic organisms as dissolution of CO_2_ into the world’s waterways continues to decrease environmental pH—a process commonly termed ocean acidification^8^. While most animals fully compensate for environmental hypercapnia related extracellular acid–base disturbances, the mechanism and the necessary energy reallocation to do so is believed to cause a variety of downstream consequences^20^. Several studies have identified an overarching consequence of future ocean conditions to be caused by energetic reallocation to promote maintenance of acid–base homeostasis and survival—reducing the capacity of organisms to grow, reproduce, and potentially survive^21–25^. In the Tanner crab, *Chionoecetes bairdi*, energy used to restore acid–base status comes at the cost of hemocyte mortality reducing the crabs ability to fight of pathogens^26^. Additionally, marine calcifying organisms, particularly those who are sessile such as bivalves and corals, generally experience reduced growth with the latter remaining functional yet ‘naked,’ as it loses its exoskeletal support^27^. Calcifying sea urchin larvae, *Strongylocentrotus droebachiensis*, experience reduced growth and development even under mild acidification^28,29^ due to loss of energy reserves following maintenance of intracellular pH^30^.

Behavioral consequences of ocean acidification have also been documented in vertebrate and invertebrate species. Fishes and elasmobranchs may experience detrimental effects including reduced olfactory sensation and increased predation^24,31^. In hermit crabs, *Pagurus bernhardus*, exposure to acidified conditions reduces their capacity to assess shell quality and take them more time to switch shells due to an overall reduction in movement^32^. In the mud crabs, *Panopeus herstii*, acidified conditions result in a reduction in their foraging ability, experiencing a reduced capacity to locate, handle, and consume oysters^33^. While these studies provide insights and potential physiological effects of global change, they struggle to provide comprehensive information as to an adapted animals’ physiological state due to the fact that in these studies, animals have not experienced generations of such exposure.

Vent endemic organisms represent one of few natural scenarios to investigate how chronic stress such as hypercapnia may drive physiological adaptations. Research has largely focused on adaptations deep-sea vent endemic animals have developed in relation to temperature, oxygen-uptake, and sulfide/hydrogen sulfide toxicity with few studies considering pH or hypercapnia. Deep-sea vent endemic species such as the gutless and mouthless vestimentiferan tubeworm, *Riftia pachyptila*, often rely on symbiotic microbial relationships to thrive under the stressful conditions^34^. *Riftia pachyptila* take advantage of the high environmental concentrations of CO_2_, sulfide, and nitrates within the vent and redirect these molecules from their extracellular space into their trophosome, a highly vascularized organ housing symbionts that produce nutrients and detoxify sulfide for the host^34^. Although blood and coelomic fluid of *R. pachyptila* contains up to 4.4 kPa ${\text{P}}_{{{\text{CO}}_{2} }}$ due to diffusion from the vent environment, it maintains a relatively constant blood and coelomic pH suggesting the tubeworms effectively compensate for acid disturbances related to their symbiotic relationship^34,35^; however, their means of CO_2_ transport is poorly understood and is not linked to respiratory pigment or protein-interactions^34,36^. Unlike their deep-sea counterparts, shallow vent endemic animals are not generally symbiotrophic^37^ and may instead have to rely on their own physiological adaptations to thrive under extreme conditions. Unfortunately, these adaptations have received minimal attention despite the ease of obtaining shallow animals compared to deep-sea inhabitants.

Kueishantao Islet (121°57′E, 24°50′N) off the northeastern coast of Taiwan houses a series of shallow, 10–30-m depth, hydrothermal vents whose discharged fluids are amongst the most acidic of the known oceanic systems (Minimum pH 1.54)^38^. Surrounding waters within this 500,000 m^2^ shallow vent system are maintained at *ca*. 30 °C and approximately pH 6.50, although some reports have measured waters as acidic as pH 5.50^39,40^, due in part to the abundance of CO_2_ within discharged fluids (> 90% CO_2_)^38,41^. *Xenograpsus testudinatus*, the shallow hydrothermal vent crab, is the sole endemic metazoan found within Kuseishantao Islet’s vent system and thrives in high density (364 individuals/m^2^)^42^ where ambient pH is between 6.5 and 5.5, becoming increasing acidic when feeding near the mouths of vent chimneys^43^. While they can survive typical oceanic conditions^44,45^, they are believed to only do so during trans-vent migration where they serve as a key species to the vent systems’ energy flow^46^ and are practically absent from surrounding non-vent fisheries as by-catch despite the area’s economic activity^47^. While Hu and colleagues^44^ identified the physiological response of normal-ocean acclimated *X. testudinatus* (pH 8.0) exposed for 2-days to waters acidified to pH 6.5, they did not investigate the animal’s native state under chronic acidification, nor the potentially exacerbated acidification experienced while feeding^43^.

To date, few studies have investigated physiological adaptations of vent endemic animals to acid–base disturbances despite pH challenge being a common environmental stress of vent environments. Here we investigated the physiological plasticity of *X. testudinatus* in extreme hypercapnic vent-like environments by creating pseudo-vent conditions in the lab via CO_2_-injection into seawater (pH 6.5 with 2.8 kPa ${\text{P}}_{{{\text{CO}}_{2} }}$ or pH 5.5 with 24.6 kPa ${\text{P}}_{{{\text{CO}}_{2} }}$). To maintain a vent-like state, crabs were moved directly from capture at the vent site into pre-acidified aquaria. Physiological strategies used by *X. testudinatus* to regulate extracellular acid–base homeostasis were investigated at the whole animal level by quantification of hemolymph acid–base status and ionic composition, metabolic rate and ammonia excretion in animals acclimated at two ends of their reported vent pH range (pH 6.5 and 5.5). Additionally, at the isolated gill level, perfusions were used to assess the gill’s ability to manipulate hemolymph-like salines under vent-like hypercapnic conditions. The results of this study highlight that even though vent endemic species are less common amongst shallow vent systems, the selective pressure of intense environmental stressors has driven animals such as *X. testudinatus* to evolve unique physiological adaptations akin to those of deep-sea vent endemic species.

## Results

### Hemolymph compensatory acid–base response

**Figure 1:**
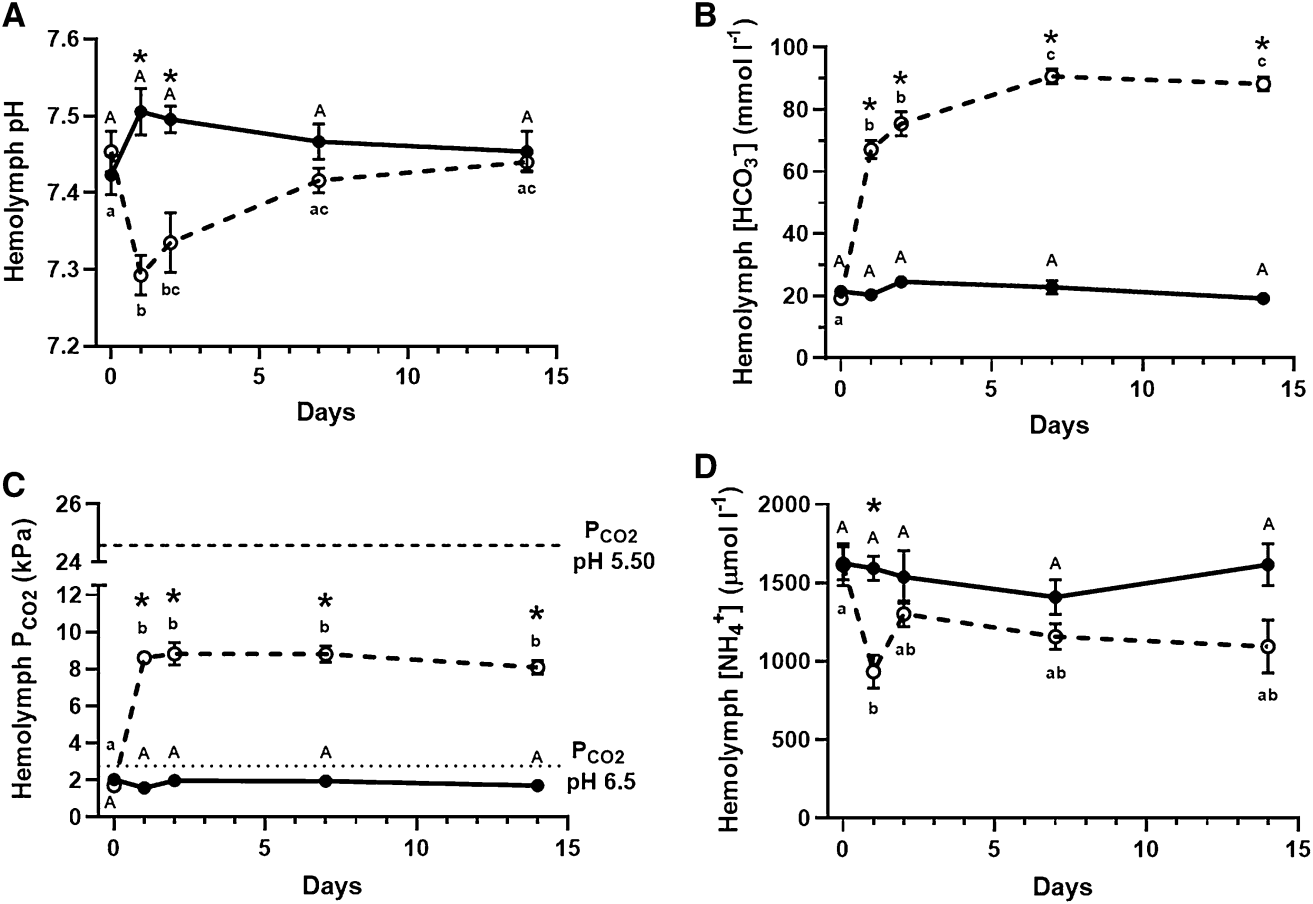
Hemolymph acid–base parameters of *Xenograpsus testudinatus* acclimated over a 14-day period to seawater acidified to either pH 6.50 (2.7 kPa ${\text{P}}_{{{\text{CO}}_{2} }}$; closed circles) or pH 5.50 (24.6 kPa ${\text{P}}_{{{\text{CO}}_{2} }}$; open circles). Changes in extracellular pH (**A**; N = 6), HCO_3_^−^ (**B**; N = 6), ${\text{P}}_{{{\text{CO}}_{2} }}$ (**C**; N = 6), and [NH_4_^+^] (D; N = 5–7) were measured from pre-branchial hemolymph after 0, 1, 2, 7, and 14 days of acclimation. Environmental ${\text{P}}_{{{\text{CO}}_{2} }}$ levels are indicated by dashed lines to indicate the presence of inwardly directed ${\text{P}}_{{{\text{CO}}_{2} }}$ gradients (**C**). Asterisks denote significance based upon acclimation pH. Upper-case letters denote time-dependent differences within pH 6.50 exposed crabs whereas lower-case letters denote time-dependent differences within pH 5.50 exposed crabs. Data presented as means ± S.E.M, *p* < 0.05.

Crabs exposed to *ca.* pH 6.5 (pH 6.49 ± 0.004, 2.77 ± 0.06 kPa ${\text{P}}_{{{\text{CO}}_{2} }}$) maintained an extracellular pH of 7.42 ± 0.03 (n = 6) throughout the time-course exposure (Fig. 1A) which is well within the normal physiological range of aquatic species. Upon exposure to *ca.* pH 5.5 (pH 5.55 ± 0.01, 24.58 ± 0.19 kPa ${\text{P}}_{{{\text{CO}}_{2} }}$), the minimum vent pH normally experienced, crabs underwent a significant respiratory acidosis (two-way ANOVA, n = 6, *p* = 0.002) marked by a. 0.16 ± 0.03 (n = 6) reduction in extracellular pH (Fig. 1A) alongside a 6.88 ± 0.34 kPa (n = 6) increase in arterial ${\text{P}}_{{{\text{CO}}_{2} }}$ (two-way ANOVA, n = 6, *p* < 0.0001; Fig. 1C). Acute compensation appears to partially rely on an increased excretion of NH_4_^+^ as a H^+^-equivalent (Fig. 1D) occurring in parallel to the accumulation of HCO_3_^−^ until the hemolymph buffering capacity is sufficient to compensate the influx of H^+^ from the environment (Fig. 1B). Extracellular pH is restored within 7-days and maintained after 14-days of acclimation (Fig. 1A) at which point the investigated hemolymph parameters stabilize – most notably when HCO_3_^−^ accumulates to 88 ± 2.2 mmol l^−1^ (n = 6) in an apparent exchange for Cl^−^ (Table 1) and as hemolymph ammonia concentrations are partially restored to control values representing a new homeostatic plateau (Fig. 1D).

**Table 1:** Major hemolymph cation and anion composition of freshly captured wild *Xenograpsus testudinatus* (N = 6) as well as crabs exposed to either pH 6.50 (2.7 kPa ${\text{P}}_{{{\text{CO}}_{2} }}$; N = 6) or pH 5.50 (24.6 kPa ${\text{P}}_{{{\text{CO}}_{2} }}$; N = 6–7) for 14-days.

Exposure	Na^+^	K^+^	Ca^2+^	Mg^2+^	Cl^−^	HCO_3_^−^	T_AMM_
pH 6.50	440 ± 10	16.7 ± 0.8	24 ± 2	36 ± 1	480 ± 10	19 ± 3	1.4 ± 0.2
pH 5.50	348 ± 7	10.1 ± 0.2	15.4 ± 0.9	41 ± 2	426 ± 5	88 ± 5	1.2 ± 0.2
Wild caught (pH 6.40)	410 ± 10	16.0 ± 0.9	14.2 ± 0.9	36 ± 1	490 ± 8	N/a	N/a

Data presented as mean concentrations (mmol l^−1^) ± S.E.M; ‘N/a’ represents unmeasured parameters.

### Isolated gill acid–base regulatory capacity under exposure to chronic (pH 6.50) and acute (pH 5.50) acidification

**Figure 2:**
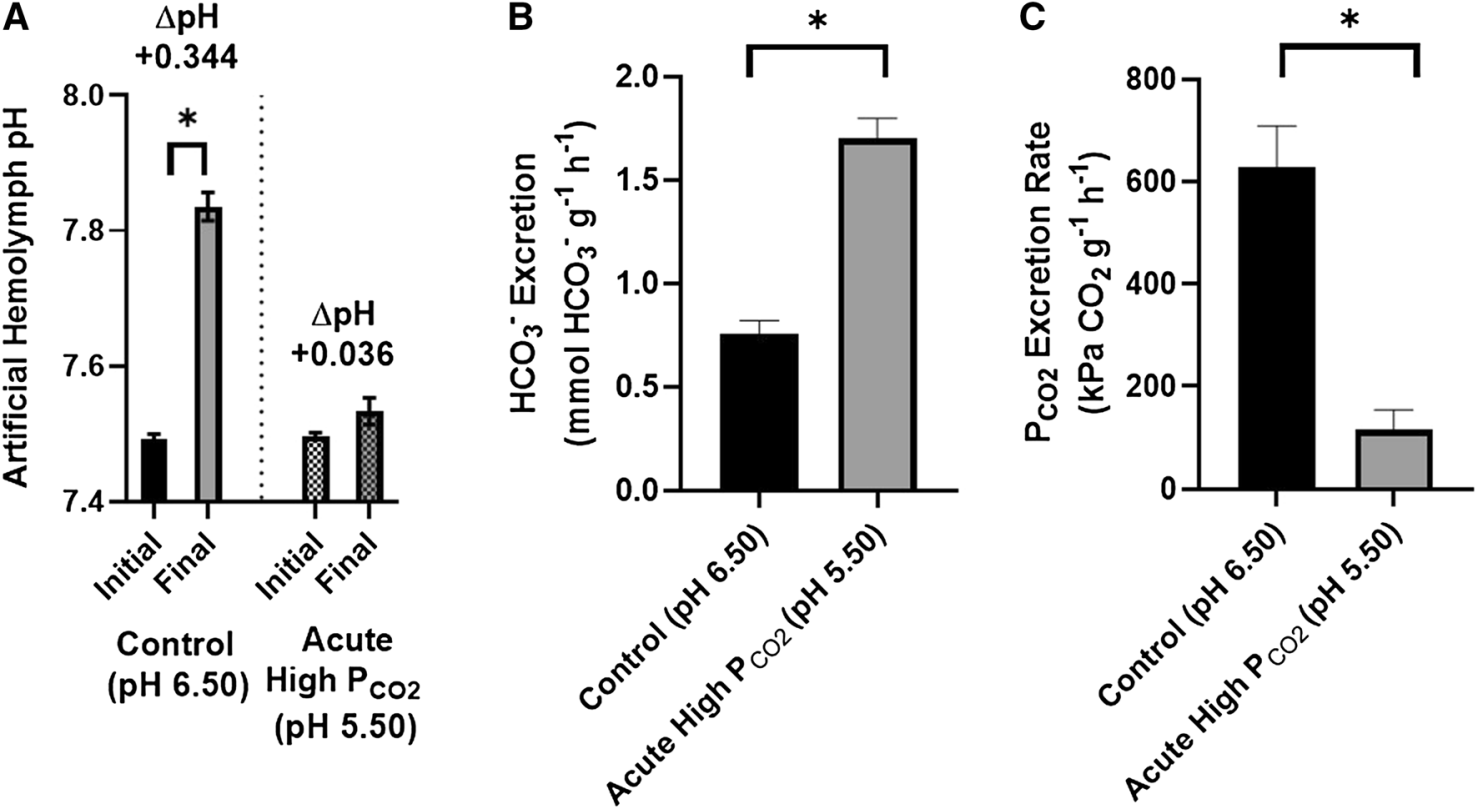
Capacity of isolated perfused gill 5 of pH 6.50 acclimated *Xenograpsus testudinatus* to alter pH (**A**), ${\text{P}}_{{{\text{CO}}_{2} }}$ (**B**), and HCO_3_^−^ (**C**) of artificial hemolymph-like saline following a single gill passage. Gills were first exposed to pH 6.50 (2.7 kPa ${\text{P}}_{{{\text{CO}}_{2} }}$) to determine their transport capacity under the acclimated condition. Gills were subsequently exposed acutely to pH 5.50 (24.6 kPa ${\text{P}}_{{{\text{CO}}_{2} }}$) to observe changes in transport capacity. Degree of hemolymph alkalization by the gill is represented as ΔpH (**A**). Positive HCO_3_^−^ and ${\text{P}}_{{{\text{CO}}_{2} }}$ excretion rates infer the molecule is lost to the environment as indicated by a lesser presence in the perfusate as compared to initial amounts within perfusion saline. Asterisks denote significance between initial and final pH or significant differences in transport rates depending on pH exposure. Data presented as means ± S.E.M, *p* < 0.05, N = 6 for all data points.

Gills of crabs fully acclimated to pH 6.5 conditions were isolated and perfused under their acclimated conditions as well as an acute exposure to increased hypercapnia. Perfusion of the fifth gill showed that the branchial tissue carries out a significant alkalinisation (ΔpH = 0.34 ± 0.01; n = 6, paired two-tailed t-test, *p* < 0.0001; Fig. 2A) of the artificial hemolymph-like saline following a single gill passage under exposure to their acclimation conditions (pH 6.49 ± 0.004, 2.77 ± 0.06 kPa ${\text{P}}_{{{\text{CO}}_{2} }}$). Increasing ambient H^+^ abundance by tenfold (pH 5.55 ± 0.01, 24.58 ± 0.19 kPa ${\text{P}}_{{{\text{CO}}_{2} }}$) reduced alkalinisation of artificial hemolymph-like saline (ΔpH = 0.036 ± 0.019; n = 6, paired two-tailed t-test, *p* = 0.11; Fig. 2A); however, the gill maintained a physiological pH of the perfusate (Fig. 1A). Interestingly, under both levels of acidification, gills were found to excrete HCO_3_^−^ (Fig. 2B) in a fashion that increased in response to acidification (n = 6, paired two-tailed t-test, *p* < 0.0001). Under acclimation conditions (*ca.* pH 6.5, ${\text{P}}_{{{\text{CO}}_{2} }}$ 2.8 kPa) it is conceivable that the gill produces excess base which is excreted or exchanged for counterions (i.e. Cl^−^). Under acute acidification (*ca.* pH 5.5, ${\text{P}}_{{{\text{CO}}_{2} }}$ 24.6 kPa), it was expected that HCO_3_^−^ would be accumulated as a compensatory mechanism which may instead be a delayed response. Excretion of CO_2_ (Fig. 2C) was maintained by the isolated gill despite a massive inward hemolymph-directed ${\text{P}}_{{{\text{CO}}_{2} }}$ gradient, suggesting an active excretory mechanism exists within the branchial tissue. Gills acutely exposed to *ca.* 24.6 kPa ${\text{P}}_{{{\text{CO}}_{2} }}$ (pH 5.5) experienced a *ca*. fourfold reduction in CO_2_ excretion rate (n = 6, pair two-tailed t-test, *p* = 0.0026) as compared to their exposure to *ca.* 2.8 kPa ${\text{P}}_{{{\text{Co}}_{2} }}$ (pH 6.5).

### Whole animal response to acidification

**Figure 3:**
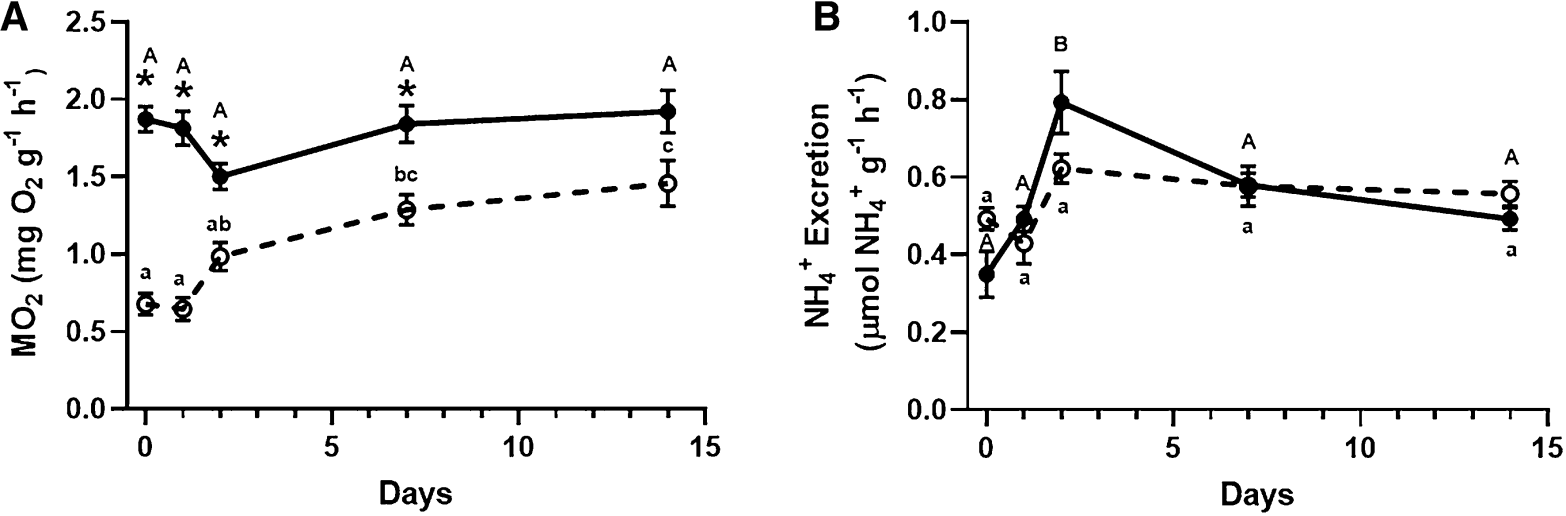
Whole animal metabolic rate (**A**) and ammonia excretion rates (**B**) were measured to indicate shifts in the crab’s metabolism in response to acidification over a 14-day time course acclimation to either pH 6.50 (2.7 kPa ${\text{P}}_{{{\text{CO}}_{2} }}$; open circles) or pH 5.50 (24.6 kPa ${\text{P}}_{{{\text{CO}}_{2} }}$; closed circles). Metabolic rate (**A**) was determined as the rate of oxygen consumption per body mass per time (µg O_2_ g^−1^ h^−1^) using closed-system respirometry (N = 6–9). Ammonia excretion rates (**B**) were determined as based on accumulation of ammonia within ambient water per body mass per time (µmol NH_4_^+^ g^−1^ h^−1^; N = 6–9). Asterisks denote significance based upon acclimation pH. Upper-case letters denote time-dependent differences within pH 6.50 exposed crabs whereas lower-case letters denote time-dependent differences within pH 5.50 exposed crabs. Data presented as means ± S.E.M, *p* < 0.05.

Metabolic rate was maintained throughout the 14-day time-course exposure to *ca.* pH 6.5 (2.8 kPa ${\text{P}}_{{{\text{CO}}_{2} }}$) at 1.87 ± 0.08 (n = 8–9) mg O_2_ g^−1^ h^−1^, which was immediately reduced by nearly 64 ± 4% (two-way ANOVA, n = 8–9, *p* < 0.0001) upon exposure to elevated hypercapnia (*ca.* pH 5.5, 24.6 kPa ${\text{P}}_{{{\text{CO}}_{2} }}$). Restoration of metabolic rate occurred over the 14-day exposure to *ca.* pH 5.5 and was statistically the same as the rate of pH 6.5-exposed crabs after 14-days (Fig. 3A). Whole animal NH_4_^+^ excretion was found to be independent of crabs’ acclimation to environmental pH (two-way ANOVA, n = 6–9, *p* = 0.38; Fig. 3B).

## Discussion

Under typical-vent conditions for *X. testudinatus*’ habitat (${\text{P}}_{{{\text{CO}}_{2} }}$ 2.7 kPa, pH 6.5), crabs maintain extracellular acid–base homeostasis (Fig. 1A) by maintaining *ca*. 20 mmol l^−1^ hemolymph HCO_3_^−^ concentration and responds to acute hypercapnia by further accumulating hemolymph HCO_3_^−^ (Fig. 1B). While this study and the sole previous study regarding acid–base capabilities of *X. testudinatus* both found that *X. testudinatus* maintains its hemolymph pH at approximately 7.40–7.50, which is considerably lower than most marine decapod crustaceans (*Metacarcinus magister* pH_e_ = 7.93^48^; *Carcinus maenas* pH_e_ = 7.87^49^; *Necora puber* pH_e_ = 7.90^50^; *Callinectes sapidus* pH_e_ = 8.00^51^), the crabs resting HCO_3_^−^ differed^44^. The resting concentration of hemolymph HCO_3_^−^ of crabs acclimated to pH 6.50 in this study is about 10 mmol l^−1^ lower than those reported by Hu and coworkers^44^. However, in the previous study, crabs were moved from long-term holding in normal seawater (pH 8.10) and moved for up to 48 h to acidified conditions (pH 6.5). If crabs had been given longer to reach a steady-state in pH 6.5 it is likely that their hemolymph HCO_3_^−^ would slightly decrease and be within the range reported in this study. This type of initial over-compensatory HCO_3_^−^ accumulation followed by a reduced plateau has been demonstrated in *Necora puber* exposed to hypercapnic seawater of pH 6.74^50^. *Necora puber*, much like *X. testudinatus*, responds to acute hypercapnia by accumulating 29.3 mmol l^−1^ HCO_3_^−^ within its hemolymph after 48 h of exposure; however, prolonged acclimation causes the crabs to reduce circulating [HCO_3_^−^] to 22 mmol l−1^50^. Acute exposure to a tenfold increase in acidity (${\text{P}}_{{{\text{CO}}_{2} }}$ = 24.6 kPa, pH 5.5) caused a respiratory acidosis (Fig. 1 A, C) that induced rapid accumulation of hemolymph bicarbonate (*ca.* 350% increase) within 24-h (Fig. 1B), amongst the highest hemolymph HCO_3_^−^ concentration ever reported for aquatic species including that of the Pacific hagfish at 6 kPa ${\text{P}}_{{{\text{CO}}_{2} }}$ for 96 h 78.2 ± 4.5 mmol l^−1^ plasma HCO_3_−^15^ or *Necora puber* exposed to 6.04 kPa ${\text{P}}_{{{\text{CO}}_{2} }}$ that accumulated 55.9 mmol l^−1^ HCO_3_^−^ after 24 h of exposure, although this exposure reported 100% mortality to the crabs after 4 days^50^. This hyperbolic increase of hemolymph HCO_3_^−^ is nearly identical to that observed by Hu and coworkers^44^ when *X. testudinatus* were acutely challenged by exposure of pH 6.5 suggesting the crab is using a similar mechanism at a greater magnitude. Hemolymph pH was fully restored after 7-days of acclimation to pH 5.5 (24.6 kPa ${\text{P}}_{{{\text{CO}}_{2} }}$; Fig. 1A) at which point [HCO_3_^−^] exceeded 90 mmol l^−1^, a response that was maintained throughout the remainder of the 14-day acclimation (Fig. 1B). The origin of HCO_3_^−^ accumulated by aquatic crustaceans has received some debate over recent years whether it is environmentally sourced, the result of carapace dissolution, or a mixture of the two. Dissolution of the carapace has been largely disproven to be a major source of HCO_3_^−^ in crabs exposed to hypercapnia as studies indicate extracellular concentrations of Ca^2+^ and Mg^2+^, the main divalent components of the exoskeleton, do not match the rise in extracellular HCO_3_^−^ concentration as would be expected^50^. Studies on *Callinectes sapidus* favour the hypothesis that accumulated HCO_3_^−^ is due to ion exchange within the gill epithelia during hypercapnic exposure based on reduced chloride uptake rates^51^. Similarly, *Riftia pachyptila* are believed to rely on diffusion of CO_2_ gas into their extracellular fluid in order to sequester carbon into their trophosome for symbionts ^34^. In this study, hemolymph ion composition indicated that HCO_3_^−^ accumulation was equally matched by the disappearance of hemolymph chloride ions (Cl^−^; Table 1) suggesting the observed accumulation of HCO_3_^−^ is from the environment as either HCO_3_^−^ directly or by rapid hydration of CO_2_ following its diffusion into the tissues. Presence of an unidentified HCO_3_^−^/Cl^−^ exchanger within the branchial tissue of *X. testudinatus* was previously hypothesized to be a major factor contributing to the crab’s strong acid–base regulatory capacity^44^. The previous study hypothesized that an increase in the enzyme activity and mRNA expression of basolateral Na^+^/K^+^-ATPase within the gills of *X. testudinatus* helped fuel HCO_3_^−^ uptake similar to that of *Neohelice granulate*^52^ and the tenidap-sensitivity of *C. maenas*’ branchial CO_2_ and H^+^ flux^49^. Pacific hagfish, referred to as the champions of CO_2_-tolerance, are the only other known water-breathing organism capable of accumulating and sustaining nearly 100 mmol l^−1^ HCO_3_^−^ within their extracellular space in response to hypercapnic exposure and is believed to do so via HCO_3_^−^/Cl^−^ exchange^15^. Despite their high capacity for CO_2_-tolerance, hagfish experienced nearly one full unit reduction in extracellular pH upon exposure to approximately 6 kPa ${\text{P}}_{{{\text{CO}}_{2} }}$ ^15^ whereas *X. testudinatus* experiences only a maximum 0.2 pH unit reduction of extracellular pH (Fig. 1A) upon exposure to 24.6 kPa ${\text{P}}_{{{\text{CO}}_{2} }}$ inferring the crab’s extraordinary acid–baseregulatory capacity. Given that hagfish are evolutionarily ‘ancient’^15^ and vent environments are ‘origin of life’ environments^53^; this high capacity of HCO_3_^−^ accumulation and/or excess Cl^−^ exchange may have been a mechanism shared amongst organisms that was later reduced in capacity as typical aquatic environments do not require such a degree of specialization.

Following the central paradigm behind animals’ respiratory physiology, CO_2_ is excreted to the ambient environment down a ${\text{P}}_{{{\text{CO}}_{2} }}$ diffusion gradient, where tensions decrease from the cellular level to the extracellular space, and eventually the environment through an excretory epithelium (i.e. lungs, gills, skin). Failure to excrete metabolic and/or environmentally sourced CO_2_ will cause animals’ vital fluids to continuously acidify until their demise. Under both hypercapnic vent conditions, environmental ${\text{P}}_{{{\text{CO}}_{2} }}$ of 2.8 kPa and 24.6 kPa, *X. testudinatus*’ hemolymph ${\text{P}}_{{{\text{CO}}_{2} }}$ was found to be maintained consistently and dramatically below environmental levels by *ca.* 0.9 kPa and 16 kPa, respectively (Fig. 1C), representing an inwardly-directed ${\text{P}}_{{{\text{CO}}_{2} }}$ gradient. Presence of an inwardly directed ${\text{P}}_{{{\text{CO}}_{2} }}$ gradient infers excretion of CO_2_ occurs either (a) across an organ whom is not exposed to the environment (i.e. antennal gland) or (b) via active-transport of CO_2_ or an equivalent molecule such as HCO_3_^−^ or CO_3_^2−^. Perfusion of isolated gills excised from crabs acclimated to pH 6.5 revealed that the branchial tissue alone excretes 628 kPa g gill^−1^ h^−1^ CO_2_ against a 2.7 kPa environmental ${\text{P}}_{{{\text{CO}}_{2} }}$ as well as 116 kPa g gill^−1^ h^−1^ upon acute exposure to 24.6 kPa ${\text{P}}_{{{\text{CO}}_{2} }}$ (Fig. 2C), suggesting the requirement of a specialized ‘concentrating’ organ unexposed to the environmental conditions, such as the antennal gland, is not necessarily required. This branchial CO_2_-excretory system, while mechanistically unclear, alkalizes hemolymph-like saline by *ca.* 0.344 pH units in a single gill passage in pH 6.5-acclimated animals while maintaining physiological pH despite acute exposure to a tenfold increase in H^+^ abundance and 22 kPa ${\text{P}}_{{{\text{CO}}_{2} }}$ (Fig. 2A). Interestingly, acute exposure to pH 5.5 resulted in an increased rate of HCO_3_^−^ loss to the environment but did not result in failure of the gill to restore homeostatic pH (Fig. 2B), suggesting an alternative acid–base equivalent, e.g. ammonium, may be important in acute response to acidification.

Active transport of CO_2_ represents a puzzling energetic challenge as the production of energy required to continually excrete mass-amounts of CO_2_ is synonymous with the production of metabolic CO_2_. In parallel, branchial epithelia must be either highly gas-selective or semi-impermeable to gases to prevent the influx of environmental ${\text{P}}_{{{\text{CO}}_{2} }}$ as well as preventing back-flow of excreted carbon, as the release of ionic CO_2_ equivalents (HCO_3_^−^ or CO_3_^2−^) will revert to CO_2_ in the acidic environment (pKa HCO_3_^−^ = 10.3). While unspecific to gases, gills of marine crustaceans are typically ion ‘leaky’^54^ which further raises required effectiveness of *X. testudinatus’* branchial capacity to maintaining physiological pH during isolated and acute exposure to a tenfold increase in H^+^ abundance and 22 kPa increase in ambient ${\text{P}}_{{{\text{CO}}_{2} }}$ (Fig. 2A). Branchial gas-selectivity or impermeability is a tempting hypothesis, while uninvestigated, it would be an asset to survival in vent-environments as it would not only explain some degree of the continual excretion of CO_2_ despite the presence of an inwardly-directed ${\text{P}}_{{{\text{CO}}_{2} }}$ gradient, but also improve *X. testudinatus*’ hydrogen sulfide resistance alongside bacterial symbiosis^55^. Gas permeability in hydrothermal vent species has only been investigated in *Riftia pachyptila* in regards to the diffusion of both H_2_S and CO_2_ into the animal’s extracellular space. Although CO_2_ was shown to freely diffuse into extracellular fluid of *Riftia pachyptila*^35^, the major uptake of sulfides by the tubeworm has been identified as uptake of HS^−^ and that diffusion of H_2_S into the animal does not occur freely^56^. Selective pressure on vent endemic species that are continuously exposed to high levels of CO_2_ and H_2_S gases may have caused some to develop a sort of semi-impermeable or selectively-permeable gas epithelia; however, there has yet to be experiments to directly prove this can occur let alone a descriptive mechanism of how it could occur. If this theory was true, the animal would require a means of efficient oxygen uptake from a relatively hypoxic or anoxic vent environment to support the animals’ metabolic rate (Fig. 3A), which is 2.5-fold higher than those of reasonably active aquatic crustaceans such as *Penaeus japonicus* (0.753 mg O_2_ g^−1^ h^−1^, 30 °C, 35 ppt) ^57^ and up to 14-fold higher than some brachyuran crabs namely the South American rainbow crab *Neohelice granulata* (0.136 mg O_2_ g^−1^ h^−1^, 21.6 °C, 2 ppt)^58^ and the blue crab *Callinectes sapidus* (0.129 mg O_2_ g^−1^ h^−1^, 30 ppt, 24 °C)^59^. Furthermore, normally negligible manipulation of extracellular non-bicarbonate buffering systems may be invaluable for these animals as transport of acid–base equivalents, such as ammonium (NH_4_^+^) that is easily excreted in acidic ammonia-free waters, may be more effective than directly manipulating components of the carbonate system. While these membrane properties are unlikely to exist in non-vent endemic species, maintenance of an inwardly directed ${\text{P}}_{{{\text{CO}}_{2} }}$ gradient while excreting CO_2_, prevention of sulfur toxicity, as well as specialized O_2_-uptake are physiological processes that would seem favorable for animals endemic to vent environments.

While the carbonate system is undoubtedly the major buffering agent within *X. testudinatus*’ hemolymph, crabs were found to maintain hemolymph ammonia *ca.* 8 to 15-fold higher (Fig. 1D) than most aquatic crustaceans^60,61^, despite ammonia’s immense toxic potential^62^. Undetectable concentrations of ambient ammonia (≤ 5 µmol l^−1^) within the vents as well as their acidic nature (Table 2; pKa ammonia *ca.* 9.25) should facilitate diffusion of NH_3_ to the environment given the presence of a *ca*. 798 Pa ${\text{P}}_{{{\text{NH}}_{3} }}$ outwardly-directed diffusion gradient and that acid-trapping of NH_4_^+^ is possible within vents, unlike slightly-alkaline marine environments whose buffering potential is believed to counteract maintenance of an acidic zone across the branchial epithelia^63^. In fact, whole animal ammonia excretion rates (Fig. 2b) were unaltered under increasingly acidified conditions as compared to control measurements and are within the range of non-vent dwelling aquatic crustaceans, such as the green crab *Carcinus maenas* (187 nmol gFW^−1^ h^−1^), the Dungeness crab *Metacarcinus magister* (367 nmol gFW^−1^ h^−1^), or the Chinese mitten crab *Eriocheir sinensis* (123 nmol gFW^−1^ h^−1^)^54,61,64^ with circulating ammonia that typically do not exceed 160 µmol l^−1^. Retention of ammonia and potential ‘ammonia homeostasis’ has been hypothesized as an important acid–base regulatory process that occurs in parallel to HCO_3_^−^ accumulation in some invertebrates such as the American horseshoe crab^65^ and the common octopus^66^. Selective retention or excretion of ammonia would require a bi-directional mechanism to be present such as the vesicular trafficking mechanism believed to be generally employed by other marine invertebrates and animals inhabiting buffered environments^19,67–69^. The vesicular trafficking mechanism hypothesizes that ammonia becomes trapped as NH_4_^+^ in vesicles acidified by either V^+^-type H^+^-ATPases (HAT) or Na^+^/H^+^ exchangers and moved within the cell by the microtubular network^69^. While this study did not test the presence of this mechanism, previous studies have localized HAT within the cytosol with minor presence along the apical membrane of *X. testudinatus*’ branchial epithelium^44^. Furthermore, HAT mRNA expression as well as its enzyme activity increases upon *X. testudinatus*’ exposure to acute hypercapnia as would be expected if the crab was to mobilize retained NH_4_^+^ as an excretable H^+^-equivalent^44^. Reliance on a typically negligible extracellular buffering agent, such as ammonia, may become more critical under situations where the movement of H^+^ or HCO_3_^−^ is less favorable as may occur when animals are abruptly challenged by a tenfold increase in ambient protons as presented in this study. Furthermore, it has been suggested that under severe acid-stress aquatic animals may preferentially degrade HCO_3_^−^ yielding amino acids such as asparagine, glutamine, and their dicarboxylic acids^70^. Such a reliance would cause an increase in ammonia and HCO_3_^−^ that could be used by *Xenograpsus testudinatus* to regulate the organism’s acid–base status by retention of HCO_3_^−^ and excretion of NH_4_^+^ as an acid-equivalent.

**Table 2:** Water physiochemical parameters of acclimatory pH 6.50 (2.7 kPa ${\text{P}}_{{{\text{CO}}_{2} }}$) and pH 5.50 (24.6 kPa ${\text{P}}_{{{\text{CO}}_{2} }}$) tanks over the 14-day acclimatory periods as well as water samples obtained during animal collection at the vent site (N = 8).

Environment	pH	Temperature (°C)	Total alkalinity (µmol kg^−1^ l^−1^)	C_T_ (µmol l^−1^)	${\text{P}}_{{{\text{CO}}_{2} }}$ (kPa)	[HCO_3_^−^] (µmol l^−1^)	T_AMM_ (µmol l^−1^)
Control	6.489 ± 0.004	30.15 ± 0.07	2361 ± 42	3020 ± 48	2.765 ± 0.056	2331 ± 40	N/a
High ${\text{P}}_{{{\text{CO}}_{2} }}$	5.549 ± 0.006	30.62 ± 0.05	2373 ± 25	8379 ± 53	24.581 ± 0.187	2374 ± 25	N/a
Vent site	6.40 ± 0.02	N/a	2475 ± 45	3365 ± 59	3.624 ± 0.135	2462 ± 44	≤ 5

Acclimatory tank conditions were monitored every 1–2 days throughout experimentation. Data presented as means ± S.E.M; ‘N/a’ represents unmeasured parameters.

Although this study identifies the surface of what physiological adaptations can be present in shallow hydrothermal vent endemic animals, the study of solely hypercapnia cannot be used as an explicative alone. While temperature could affect the observations made in this study, vent inhabitants may correct for variations by repositioning their proximity to the vents. Some stressors, however, are more difficult for animals to respond to. For example, high environmental sulfide levels at Kuieshantao Island’s vent fields may have a substantial impact on their metabolic rate and thus their ability to recover from environmental challenges, particularly acidification due to the pH-sensitivity of sulfide toxicity. Sulfide’s expected inhibition of cytochrome C oxidase and resultant disruption of ATP synthesis^71^ would be expected to severely impact the energetic demand *X. testudinatus* is expected to require in order to rapidly accumulate HCO_3_^−^. Further investigations into the individual affects of sulfur as well as observing the crab’s response to a combination of hypercapnia and sulfides would be of interest, especially given the permanent presence of both in their native environment.

While it is clear that *X. testudinatus* has developed unique adaptations to thrive in its environment, it remains unclear what evolutionary history the crab may have experienced in order for them to develop. While it may be appealing to hypothesize that the physiology of *X. testudinatus*, and other vent endemic species, is due to it being an ‘antique’ species or ‘living fossil,’ molecular evidence has indicated that modern vent endemic fauna are relatively recently diversified as recently as the last 100 million years (see review by Van Dover and coworkers^72^ and Little and Vrijenhoek^73^). In fact, modern deep-sea and seep fauna are believed to have originated from environments such as shallow hydrothermal vents^73^. This raises the question of whether the physiological achievements of deep-sea fauna, approximately 10% of which are decapod crustaceans^74^, are similar or based upon those of species like *X. testudinatus* that have since taken refuge in deeper environments over generations. Unfortunately, studies of shallow vent endemic fauna have not received the attention that deep-sea vent have regarding crustaceans. Transcriptomic information has only recently begun to exist for vent endemic crustaceans in regards to how physiological adaptations have originated, although only in deep-sea species^75^. Further evolutionary investigation into shallow vent endemic fauna and its pairing to physiological measurements may help answer questions related to hard-to-obtain deep-sea counterparts.

Cumulatively, the reported physiological results may reflect the physiological plasticity developed by *X. testudinatus* in response to how their feeding behaviour relates to harsh conditions created by vent emissions. At high and low tides during feeding, *Xenograpsus testudinatus* approaches the mouths of vents to feed upon zooplankton killed by the hot, sulfuric, and acidic vent emissions^43^, exposing the animals to an acute increase in acidity, hypoxia, hypercapnia, and high levels of sulfides. The observed immediate 64% reduction in metabolic rate upon exposure to pH 5.5 (24.6 kPa ${\text{P}}_{{{\text{CO}}_{2} }}$) may indicate that crabs reduce their ventilatory rate upon exposure as to minimize the exchange of hazardous waters over the gills – similar to how terrestrial and semi-terrestrial crabs may retain fluids within their branchial chambers^76^. As metabolic rate and cardiac output positively correlate in some animals^77–80^, hemolymph flow rates throughout the crab may slow upon acute exposure to hypercapnia. Reduced branchial perfusion rates may allow for a more thorough exchange of HCO_3_^−^/Cl^−^ to occur across the gills, forming post-branchial hemolymph with a greater buffer capacity to wash over the animals’ tissue. Furthermore, while feeding *X. testudinatus* should experience an increase in hemolymph ammonia due to protein catabolism. Experimental results indicate that following 24-h of exposure to increased acidity, hemolymph ammonia is reduced by 41% (Fig. 1D) but was in the following days restored as acid–base homeostasis returned. As a marine crustacean, *X. testudinatus*’ gill epithelia are expected to be ion “leaky”^54^, facilitating likely the influx of H^+^ through paracellular junctions upon acute exposure to increasing environmental acidity. Paracellular ion fluxes may then be reduced by increasing epithelial tightness following sufficient acclimation time, similar to how tissue conductance changes under salinity stress in crustaceans^81,82^. Vent mouths, where emitted fluids may be pH 1.52^38^, may create inwardly directed H^+^ gradients too large to effectively rely on H^+^ extrusion or HCO_3_^−^ accumulation alone. Dietary ammonia, as well as stores within the hemolymph, may be protonated forming ammonium and actively excreted^83,84^ within proximity to vents along the ammonia concentration and ${\text{P}}_{{{\text{NH}}_{3} }}$ gradients. While unmeasured, transbranchial NH_3_ and/or NH_4_^+^ excretion and retention may be partially responsible for the restoration of hemolymph pH (Fig. 1A), despite increased transbranchial HCO_3_^−^ excretion in perfused gills acutely exposed to pH 5.5 (Fig. 2B), a pH regime crabs could experience during feeding at closer proximities to the vent. In addition to the increased acidity associated with feeding, *X. testudinatus’* may experience acid-stress due to the affects of ocean acidification. Many of the aquatic animals expected to be heavily impacted by ocean acidification are those that inhabit niche zones as they may already be operating at or near maximal physiological capacity. While *X. testudinatus* does inhabit a niche zone, it is capable of surviving a broad pH range (pH 8.2–5.5) based on results of this study as well as experiments by Hu et al.^66^ and its known migration through non-vent zones while seeking neighboring vent fields ^47^. Furthermore, vent conditions near Kuieshantao already surpass the maximal projected ${\text{P}}_{{{\text{CO}}_{2} }}$ and pH values given by the IPCC even if considering the worst case ‘business-as-usual’ scenario^8^. As such, it seems illogical that the vent environment would become more acidic, as an increase in the ${\text{P}}_{{{\text{CO}}_{2} }}$ of surrounding surface waters would only reduce the rate that CO_2_ diffuses from the vent site. In essence, the normal pH zone *X. testudinatus* inhabits, *ca.* pH 6.5, would become larger in diameter but not become more acidic. Given the animal’s strong acid–base regulatory capacity and the described assumptions regarding ocean acidification’s affect on Kueishantao Island’s vent fields it seems *X. testudinatus* will not experience great consequences due to ocean acidification.

Investigating the physiological states and responses of vent-endemic species offers valuable information as to how animals may be required to change biologically in response to future changes in ocean physiochemistry. Maintenance of extracellular acid–base homeostasis within *X. testudinatus* appears to rely on conventional compensatory methods that operate at a vastly amplified capacity. These amplified compensatory methods, and the animal’s ability to maintain an extracellular ${\text{P}}_{{{\text{CO}}_{2} }}$ less than its ambient environment, would be beneficial when crabs are in close proximity to the vent emissions as may occur when feeding^43^. While it is unlikely that the majority of animals are capable of maintaining an inwardly directed ${\text{P}}_{{{\text{CO}}_{2} }}$ gradient, measuring the ability of aquatic species to perform HCO_3_^−^/Cl^−^ exchange and their capacity to manipulate non-bicarbonate buffering systems holds value in assessing their future status. In *X. testudinatus*, a large degree of extracellular chloride is replaced with accumulated HCO_3_^−^ (Fig. 1B and Table 1) exceeding the apparent limits of the bicarbonate concentration threshold described by Heisler^85^, potentially indicated vent endemic animals cope with acid-stress by carrying an expendable excess of Cl^−^. Non-bicarbonate buffering systems, such as ammonia, may become more crucial to maintaining physiological pH due to their independence from the carbonate equilibrium and ${\text{P}}_{{{\text{CO}}_{2} }}$.

## Methods and methods

### Animals and acclimatory conditions

Adult *X. testudinatus* were captured from Kueishantao’s vent field (121°57′E, 24°50′N) and either directly sampled for hemolymph parameters or transported to pre-acidified acclimation tanks within the Institute of Cellular and Organismal Biology’s Marine Research Centre. Water samples collected in parallel indicated the native environment of these crabs was similar to experimental pH 6.50 conditions (Table 2). Water samples were obtained in duplicate by divers using sealed vessels with one sample being measured for pH immediately upon resurfacing. Upon returning to the research station, both the previously measured and un-opened remaining samples were measured for pH, total carbon, and ammonia (see methods below).

Six aquaria (3 × pH 6.50 and 3 × pH 5.50; 75 L, 20–24 crabs per tank) operated as flow-through systems (flow of *ca.* 3 l h^−1^) fed directly with natural seawater (35 ppt). Animals were selected at random from tanks for all experiments and permanently removed from the experimental tanks after sampling. Aquaria were set to mimic the normal-to-highest reported acidities the crabs normally inhabit—namely pH 6.5 and 5.5, respectively—as adjusted by CO_2_-injection (pH controller, MACRO) for either long-term acclimation (pH 6.5) or 14-days in elevated ${\text{P}}_{{{\text{CO}}_{2} }}$ (pH = 5.5), as detailed in Table 2. Temperature was maintained constant at 30 °C throughout all trials as was a 12:12 h light:dark cycle. All crabs were given an initial 14-days to pre-acclimate to the control conditions and to adjust following removal from their native environment. A total of 143 crabs were used throughout the experiment, 61 of which were used exclusively for hemolymph measurements (30 crabs in pH 6.5 and 31 crabs in pH 5.5) and 82 of which were used for whole animal experiments (43 crabs in pH 6.5 and 39 crabs in pH 5.5).

Whole animal experiments and hemolymph sampling were not assessed using repeated measures. Crabs were measured for metabolic rate and ammonia excretion rate, and then returned to their acclimatory conditions for approximately 3-h prior to hemolymph sampling to allow hemolymph to stabilize due to handling stress. Sampled crabs were then removed from the pool of experimental animals and not reused at additional time points. Crabs were fed diced squid ad libitum every two days but were fasted for a minimum of two days prior to all experimentation.

### Hemolymph and water sampling

Hemolymph was sampled and analyzed following methods described by Hu et al.^44^ where samples were maintained at 30 °C for all measurements using a temperature-controlled water bath. Extracellular pH was measured immediately using an InLab Micro Combination pH electrode (Mettler-Toledo, Greisensee, Switzerland) attached to a pH-ISE meter model 225 (Denver Instruments, Gottingen, Germany). The pH was measured in the NBS scale and electrodes calibrated with pH buffers (4.01, 7.00, 10.01) traceable to NIST standard reference material (Thermofisher Orion). Sub-samples were then diluted 1:2 with crab ringer without HCO_3_ (see gill perfusion for ion composition) and their total dissolved inorganic carbon (C_T_) was measured in duplicate using a Corning 965 carbon dioxide analyzer (Olympic Analytical Services, England). Hemolymph ${\text{P}}_{{{\text{CO}}_{2} }}$ was mathematically determined using a rearrangement of the Henderson-Hasselbalch equation (Eq. 1) and *Carcinus maenas*’ carbonate system pK’ and αCO_2_ constants derived by Truchot ^86^ adjusted for temperature and osmolality of the hemolymph (pK1 = 5.807273697, αCO_2_ = 0.000254931 mmol l^−1^ Pa^−1^).

1 $$ P_{CO2} = {{TCO_{2} } \mathord{\left/ {\vphantom {{TCO_{2} } {\left[ {\left( {1 + antilog\left( {pH - pK^{\prime}} \right)} \right)*\alpha CO_{2} } \right]}}} \right. \kern-\nulldelimiterspace} {\left[ {\left( {1 + antilog\left( {pH - pK^{\prime}} \right)} \right)*\alpha CO_{2} } \right]}} $$

Hemolymph [HCO_3_^−^; mmol l^−1^] was then further calculated using Eq. 2:

2 $$ \left[ {HCO_{3}^{ - } } \right] = TCO_{2} - \left( {P_{CO2} *\alpha CO_{2} } \right) $$

The remaining undiluted hemolymph samples were frozen and stored at − 80 °C for the measurement of ammonia concentration and major ion composition. Measurement of pH and C_T_, as well as storage of water samples, was similarly performed and analyzed by the same methods as described above, however, C_T_ was determined using a Corning 965 total carbon dioxide analyzer as well as a Dissolved Inorganic Carbon Analyzer [Model AS-C3; Apollo SciTech) and a Li-7000 CO_2_/HCO_3_^−^ Analyzer (LiCOR)] for verification. Seawater HCO_3_^−^ and ${\text{P}}_{{{\text{CO}}_{2} }}$ was calculated directly inputting measured C_T_, pH, temperature, and salinity into CO2SYS software (Lewis and Wallace 1998) using dissociation constants of Mehrbach et al.^87^ refit by Dickson and Millero^88^. Ammonia concentrations of hemolymph and water samples were determined using the amino acid and protein insensitive p-orthophtaldialdehyde method described by Holmes et al.^89^ using a microplate reader (Molecular Device, SpectraMax, M5). Hemolymph and water major cation composition (Na^+^, K^+^, Ca^2+^, Mg^2+^) was determined via flame absorption spectrophotometry (Polarized Zeeman Atomic Absorption Spectrophotometer Z-5000, Hitachi High-Technologies, Tokyo, Japan), whereas Cl^−^ concentration was determined spectrophotometrically using the mercury (II) thiocyanate method adjusted for microplate use^90^.

### Whole animal ammonia excretion

Whole animal ammonia excretion rates were determined as the change in ambient ammonia concentration following a 1.5-h incubatory period to account for the stress-invoked release of ammonia. Water was carefully drained and refilled after the incubatory period via suction as to minimize animal stress and mitigate the influence of incubatory-period ammonia accumulation on ammonia excretion rates. Crabs were individually isolated inside beakers containing 75 ml of 3X-filtered (0.45 µm) seawater, pre-adjusted to the appropriate pH via CO_2_-injection. Beakers were connected to two series of polyethylene tubing capable of delivering either CO_2_ or air in order to roughly control pH and oxygenation throughout the exposure using an additional ‘proxy’ beaker fitted with a pH-probe. CO_2_ or air was manually injected if the seawater’s pH changed by *ca.* 0.3 pH units above or below acclimation conditions, respectively. Samples were collected 30, 60, and 90 min following the incubatory period and used to determine the excretion rate per crab mass as µmol NH_4_^+^ g^−1^ h^−1^.

### ***Calculation of ***${\text{P}}_{{{\text{NH}}_{3} }}$

Partial pressure of NH_3_ (${\text{P}}_{{{\text{NH}}_{3} }}$) was calculated by determining the speciation of NH_3_ and NH_4_^+^ from total ammonia (T_Amm_; µmol l^−1^) using a rearrangement of the Henderson-Hasselbalch equation (Wright and Wood^91^; Eqs. 3 and 4) where pH of either water or hemolymph and an appropriate pK’ was selected based on salinity and temperature using nomograms by Cameron and Heisler^92^. As ambient ammonia within vent fields was below detectable methods (Table 2), calculations were completed assuming 1 µmol l^−1^ T_Amm_ within the acidified waters and 1500 µmol l^−1^ within hemolymph of *X. testudinatus*.

3 $$ \left[ {NH_{4}^{ + } } \right] = {{T_{Amm} } \mathord{\left/ {\vphantom {{T_{Amm} } {\left[ {1 + antilog\left( {pH - pK^{\prime}} \right)} \right]}}} \right. \kern-\nulldelimiterspace} {\left[ {1 + antilog\left( {pH - pK^{\prime}} \right)} \right]}} $$

Speciation of NH_3_ was then determined using the following equation:

4 $$ \left[ {NH_{3} } \right] = T_{Amm} - \left[ {NH_{4}^{ + } } \right] $$

${\text{P}}_{{{\text{NH}}_{3} }}$ was then calculated by dividing the concentration of NH_3_ by αNH_3_ using the following equation in conjunction with 30 °C NH_3_ solubility coefficients (αNH_3_; µmol l^−1^ torr^−1^) derived by Boutilier et al.^93^:

5 $$ P_{NH3} = \left[ {NH_{3} } \right]/\alpha NH_{3} $$

The differential ${\text{P}}_{{{\text{NH}}_{3} }}$ gradient between the hemolymph and environment (Δ${\text{P}}_{{{\text{NH}}_{3} }}$) determined using Eq. 6 where a positive value infers an environmentally directed gradient favouring excretion of NH_3_ along its diffusion gradient.

6 $$ \Delta P_{NH3} = P_{a} NH_{3} - P_{w} NH_{3} $$

### Resting metabolic rate

Metabolic rate was measured using closed system respirometry techniques where crabs were isolated in darkened airtight glass chambers filled with 350 ml of 3X-filtered seawater (0.45 µm) pre-adjusted to the appropriate temperature and pH via CO_2_-injection. Crabs in the pH 5.50 exposure group were removed from control acclimatory tanks and immediately exposed to pH 5.50 during these experiments. Chambers were submerged in a temperature-controlled water bath to maintain a constant temperature of 30 °C. Oxygen concentrations were continuously measured using fiber optic oxygen sensors (PreSens oxygen micro optode, type PSt1; PreSens Precision Sensing GmbH, Regensburg, Germany) connected to an OXY-4 Mini multichannel fiber optic oxygen transmitter (PreSens Precision Sensing GmbH). Ventilation rate of the crabs was found to sufficiently mix water within the chamber as indicated by a linear decline in oxygen bioavailability. Oxygen consumption was determined until oxygen was approximately 60% of air saturation levels at which point crabs were weighed. Background respiration was measured in parallel to all animal measurements by measuring oxygen consumption in a similar fashion in the absence of crabs. Oxygen consumption rates were calculated as the linear decline in oxygen concentration throughout the duration of the experiment, after accounting for bacterial influence, per gram of crab per hour (µg O_2_ g^−1^ h^−1^).

### Gill perfusions

Crabs acclimated to pH 6.5 were terminated at the end of the time-course trial and their fifth gill, selected due to its feasible size. Excised gills were then submerged in hemolymph-like perfusion saline based on ion composition of their hemolymph (in mmol l^−1^: 400 Na^+^, 17 K^+^, 36 Mg^2+^, 24 Ca^2+^, 518 Cl^−^, 20 HCO_3_^−^, 1.5 NH_4_^+^; pH 7.48). Perfusion saline was further supplemented with glucose (0.3 mmol l^−1^) and L-glutamine (0.5 mmol l^−1^) as nutrient sources as well as glutathione (0.1 mmol l^−1^) to promote gill viability. Bathing solution was prepared as artificial seawater (in mmol l^−1^: 402 Na^+^, 10 K^+^, 10 Ca^2+^, 55 Mg^2+^, 547 Cl^−^, 2 HCO_3_^−^) in order to better control the ambient conditions.

Under a dissecting microscope, the afferent vessel was cannulated with FEP-tubing led into the gill by a Microloader pipette tip whereas the efferent vessel was directly cannulated with a Microloader pipette tip and collected as perfusate. Gills and the cannula were sealed using modified neoprene clamps fitted with an outer neoprene layer to ensure the gill floated without contacting surfaces. Gills were perfused in glass petri-plates containing 15 ml of pH-adjusted and filtered seawater (0.45 µM). The pH of the bathing solution was continuously monitored using a micro pH-electrode and manually adjusted by injecting either CO_2_ or air to increase or decrease acidity, respectively. Temperature was maintained at approximately 30 °C using the light source of the microscope.

Gills were allowed 15 min to equilibrate in the setup at pH 6.5 and then perfusate was collected for 20 min for the sampling period. Following measurements at pH 6.5, the gills were acutely exposed to pH 5.5 via CO_2_ injection into the bathing solution. After equilibrating for 15 min at pH 5.5, the perfusate was collected for 20 min. Perfusate pH and C_T_ was measured and ${\text{P}}_{{{\text{CO}}_{2} }}$ and [HCO_3_^−^] calculated as described for hemolymph. The capacity of the gill to alter initial perfusion saline during the passage through the gill was used to determine the acid–base transport capacities of the tissue. While perfusion describes the capacity of the gill to alter ${\text{P}}_{{{\text{CO}}_{2} }}$ and HCO_3_^−^ of artificial hemolymph-like saline in a single passage, rates were determined assuming equal transport per hour, per tissue mass.

### Statistical analysis

Statistical analyses were performed using GraphPad Prism 8.2.1 for Windows (GraphPad Software, San Diego, California USA, www.graphpad.com). Data were tested for normality using the Shapiro–Wilk test and tested for homogeneity of variance using either an F-test or Levene’s test. A two-way ANOVA was performed to analyze hemolymph acid–base parameters, whole animal ammonia excretion, and resting metabolic rate with time and pH as variables and subsequently tested using the post hoc Holm–Sidak’s multiple comparison tests. Paired *t*-tests were used to compare perfused gills ability to manipulate pH, HCO_3_^-^, and CO_2_ as well as differences between ion concentrations of hemolymph and/or seawater. Data within all texts and figures are presented as the mean ± S.E.M where significance was denoted as *p* < 0.05.

## Supplementary information

Supplementary file1 (DOCX 19 kb)

## Data Availability

All the data presented within this research article are freely available upon request to the corresponding author.
