# Supplementary material for: Specialized adaptations allow vent-endemic crabs (Xenograpsus testudinatus) to thrive under extreme environmental hypercapnia
Source: Sci Rep. 2020 Jul 16;10:11720. doi: 10.1038/s41598-020-68656-1 (PMC7367285; doi:10.1038/s41598-020-68656-1)
Supplement: Supplementary file 1 — Supplementary file1 (DOCX 19 kb) [file 41598_2020_68656_MOESM1_ESM.docx]

***Title***

Specialized adaptations allow vent-endemic crabs (*Xenograpsus testudinatus*) to thrive under extreme environmental hypercapnia.

***Authors***

Garett J.P. Allen^1^, Pou-Long Kuan^2^, Yung-Che Tseng^2^, Pung-Pung Hwang^3^, Alex R. Quijada-Rodriguez^1^, and Dirk Weihrauch^1*^

^1^Biological Sciences, University of Manitoba, 190 Dysart Rd., Winnipeg, Manitoba R3T 2M8, Canada

^2^Institute of Cellular and Organismal Biology’s Marine Research Station, Academia Sinica, No. 23-10 Dawen Rd., Jiaoxi, Yilan County, Taiwan, 262

^3^Institute of Cellular and Organismal Biology, Academia Sinica, No. 128, Section 2, Academia Rd., Nangang District, Taipei City, Taiwan, 11529

***Corresponding author***

Dirk Weihrauch, 190 Dysart Rd., Winnipeg, Manitoba R3T 2M8

Email: Dirk.weihrauch@umanitoba.ca

ORCID ID: 0000-0002-3218-9093

**Supplemental Information:**

SI 1. Results of the two-way ANOVA tests related to hemolymph pH in *X. testudinatus* exposed to hypercapnia over a 14-day time-course.

Hemolymph pH

| Source | *df* | MS | *F* | *p* |
| --- | --- | --- | --- | --- |
| Variable A (Time) | 4 | 0.004865 | 1.266 | P = 0.2957 |
| Variable B (CO_2_) | 1 | 0.09939 | 25.87 | P < 0.0001 |
| A × B | 4 | 0.03124 | 8.131 | P < 0.0001 |
| Residual | 50 | 0.003842 |  |  |

SI 2. Results of the two-way ANOVA tests related to hemolymph HCO_3_^-^ in *X. testudinatus* exposed to hypercapnia over a 14-day time-course.

Hemolymph HCO_3_^-^

| Source | *df* | MS | *F* | *p* |
| --- | --- | --- | --- | --- |
| Variable A (Time) | 4 | 2548 | 89.48 | P < 0.0001 |
| Variable B (CO_2_) | 1 | 32410 | 1138 | P < 0.0001 |
| A × B | 4 | 2524 | 88.62 | P < 0.0001 |
| Residual | 50 | 28.48 |  |  |

SI 3. Results of the two-way ANOVA tests related to hemolymph ammonia^-^ in *X. testudinatus* exposed to hypercapnia over a 14-day time-course.

Hemolymph Ammonia

| Source | *df* | MS | *F* | *p* |
| --- | --- | --- | --- | --- |
| Variable A (Time) | 4 | 302451 | 3.477 | P = 0.0138 |
| Variable B (CO_2_) | 1 | 1823475 | 20.96 | P < 0.0001 |
| A × B | 4 | 165561 | 1.903 | P = 0.1242 |
| Residual | 51 | 86996 |  |  |

SI 4. Results of the two-way ANOVA tests related to hemolymph P_CO2_^-^ in *X. testudinatus* exposed to hypercapnia over a 14-day time-course.

Hemolymph P_CO2­_

| Source | *df* | MS | *F* | *p* |
| --- | --- | --- | --- | --- |
| Variable A (Time) | 4 | 27.02 | 47.75 | P < 0.0001 |
| Variable B (CO_2_) | 1 | 430.4 | 760.6 | P < 0.0001 |
| A × B | 4 | 30.31 | 53.57 | P < 0.0001 |
| Residual | 50 | 0.5659 |  |  |

SI 5. Results of the two-way ANOVA tests related to whole animal metabolic rate in *X. testudinatus* exposed to hypercapnia over a 14-day time-course.

Whole animal metabolic rate

| Source | *df* | MS | *F* | *p* |
| --- | --- | --- | --- | --- |
| Variable A (Time) | 4 | 0.8574 | 11.54 | P < 0.0001 |
| Variable B (CO_2_) | 1 | 11.32 | 152.3 | P < 0.0001 |
| A × B | 4 | 0.6258 | 8.424 | P < 0.0001 |
| Residual | 70 | 0.07428 |  |  |

SI 6. Results of the two-way ANOVA tests related to whole animal ammonia excretion in *X. testudinatus* exposed to hypercapnia over a 14-day time-course.

Whole animal ammonia excretion_­_

| Source | *df* | MS | *F* | *p* |
| --- | --- | --- | --- | --- |
| Variable A (Time) | 4 | 0.1537 | 10.26 | P < 0.0001 |
| Variable B (CO_2_) | 1 | 0.01146 | 0.7649 | P = 0.3848 |
| A × B | 4 | 0.03249 | 2.290 | P = 0.0683 |
| Residual | 70 | 0.01498 |  |  |
